# Supplementary material for: Dormancy release of seeds of Podophyllum hexandrum Royle accompanied by changes in phytochemicals and inorganic elements
Source: PLoS One. 2023 Nov 16;18(11):e0294673. doi: 10.1371/journal.pone.0294673 (PMC10653421; doi:10.1371/journal.pone.0294673)
Supplement: Attachment — Submitted filename: Response to Reviewers.docx [file pone.0294673.s002.docx]

**Response to Reviewers**

We thank the academic editors for guidance and helpful comments. According to suggestions, we have made a lot of changes to the full text, includes abbreviation of species' name, case-sensitivity of text, standardization of geographic information, correction of clerical errors throughout the text and better description of figures.

**Academic editor Comments:**

1. L35, 36-37 and elsewhere in the text: Once fully introduced, the plant species' name should be shortened to P. hexandrum containing no authority name. Full species name can remain in its first mention in Abstract, Introduction and M&M. The same applies to L111.

**Authors’ Response:** We have completely revised the species' name in the manuscript.

2. L39-40: Please choose keywords not mentioned in the main title that are closely associated to the study theme.

**Authors’ Response:** We replaced the keywords as per the comments.

3. "Podophyllum hexandrum is a perennial Sinopodophyllum Ying herb that is..." cannot stand, since a species cannot belong to two genera. Please rewrite as suggested in the first review round..

**Authors’ Response:** Thanks for the detailed suggestions, we have made careful corrections and modifications.

4. Podophyllum hexandrum is not listed in the IUCN Red List under any threatened category. Please remove this remark.

**Authors’ Response:** Thanks for the valuable suggestion, we have removed this description.

5.L77,78, and elsewhere in the text: Please do not capitalize compound names or randomly chosen words (see the attached file where I highlighted SOME BUT NOT ALL places of intervention).

**Authors’ Response:** Thank you very much for your suggestions, we have made very careful changes!

6. L130-131: Please provide geographic coordinated in the internationally accepted format.

**Authors’ Response:** Thank you for your suggestions, we have made changes to the geographic information.

7.L177, 179: some strange signs stand here.

**Authors’ Response:** Thank you very much for your careful review, we have been carefully revised.

8.L285: What "The same as below" means?

**Authors’ Response:** Thank you very much for your careful review, we have removed this description.

9.Fig 4, Fig 5, Fig 6, Fig 7, Fig 9, Fig 10, Fig 11: Provide here informative captions. They should contain short descriptions of what can be seen from the bars, dots, etc. and description of what bars/dots were compared and their statistical significance.

**Authors’ Response:** Thanks to your valuable suggestions, we have added short informative notes and additional explanations for some of the information in the diagrams.

10.Fig 8: Please provide better descriptions of what can be seen from the graphs and provide PC components' contribution.

**Authors’ Response:** Thanks to your valuable suggestions, in order to better describe the content of the graphs, we have added explanations of the data in the graphs and added their statistical significance.
